# Supplementary material for: Protocol for chronic hepatitis B virus infection mouse model development by patient-derived orthotopic xenografts
Source: PLoS One. 2022 Feb 23;17(2):e0264266. doi: 10.1371/journal.pone.0264266 (PMC8865695; doi:10.1371/journal.pone.0264266)
Supplement: Attachment — Submitted filename: Response to Reviewers.docx [file pone.0264266.s001.docx]

Response to Reviewer #1 comments:

Dear Reviewer,

We want to thank you for the thoughtful feedback. We have incorporated all of your comments to our revision and added some points to discussion. We also provide a point-by-point reply to all of your comments.

***Comment 1:***

As is mentioned in <https://journals.plos.org/plosone/s/what-we-publish#loc-study-protocols> Study Protocols describe detailed plans for conducting research, including the background, rationale, objectives, methodology, statistical plan, and organization of a research project.

***Comment 2:***

We’ve supplemented the manuscript with graphics pipelines.

***Comment 3:***

We tried to reorganize the “Material and methods” section.

On behalf of authors, I want to thank Reviewer #1. All comments were very helpful.

Best regards,

Aleksey Nagornykh.

Response to Reviewer #2 comments:

Dear Reviewer,

On behalf of all co-authors, I thank you for your time and efforts on revising the manuscript. We now submit the revised text. We’ve carefully revised our manuscript according to your suggestions.

***Comment 1:***

We’ve adjusted the sections “Research objectives” and “Study aim”. Expected results are described in the “Discussion” section. No data sets were generated or analyzed during the current study. All relevant data of this study will be made available upon study completion. Results will be presented in one of the peer-reviewed specialized journals in accordance with the protocol described in our manuscript.

***Comment 2:***

We’ve changed Reference 5 to a more suitable one.

***Comment 3:***

We’ve included description of the humanized mice to “Introduction” section.

***Comment 4:***

That was a typo. Models of viral infections were implied. We’ve included information about the literature to “Introduction” section, references 40-44.

***Comment 5:***

That was a typo. qPCR was implied.

***Comment 6:***

We don’t plan to irradiate mice after the grafts. There is no mention of irradiating mice after grafts in our manuscript.

***Comment 7:***

We’ve included examples of literature to **“**HCC-PDX-avatars development**” section, references 56, 57.**

***Comment 8:***

We’ve supplemented “HCC-HBV-PDOX-avatars development” section with information about postoperative animal care. Humane endpoints were defined in the first version of the manuscript: “Observation of the animals is continued until the tumor reaches of 2 cm in diameter or a body weight loss of more than 10%, but not more than 2 months”. When working on the manuscript after your comment, this phrase was reformulated.

On behalf of authors, I want to thank Reviewer #2. All comments were very helpful.

Best regards,

Aleksey Nagornykh.
